# The Universal Set of 99 InDel Markers for Human Identification

**DOI:** 10.3390/biology13120993

**Published:** 2024-11-29

**Authors:** Alexander V. Chudinov, Ivan D. Ivanovsky, Sergey A. Polyakov, Alexander S. Zasedatelev, Denis O. Fesenko

**Affiliations:** 1 Engelhardt Institute of Molecular Biology, Russian Academy of Sciences, 119991 Moscow, Russia; 2 DNA Research Center, LLC, 141402 Hkimki, Russia; dnarescenter@gmail.com

**Keywords:** human identification, InDel, microarray, biochip, forensic genetics, Ecuadorian population, Russian population

## Abstract

The aim of this work was to select InDel markers sufficient for human identification and to create a routine method for their genotyping. We analyzed the allele distribution of all known InDels in European, East Asian, South Asian, African, and American populations and selected markers whose minor allele frequency, MAF, was ≥ 0.30. Thus, a set of 99 polymorphisms was formed with an average MAF for all five populations of 0.397 and a distance between markers of ≥3 million bp. The expected values of combined random match probability differ very little between the five superpopulations and are in the range of 1.44 × 10^−41^–3.68 × 10^−41^. For the genotyping of this set of markers, we have developed a method that includes single-step multiplex PCR followed by hybridization on a biochip. The average amplicon length was 72 bp. Aiming to verify the usefulness of 99 biallelic InDels in genetic identification and kinship testing, two phylogenetically distant populations were studied: Russian and Ecuadorian. For the Russian population, the combined random match probability was 2.09 × 10^−40^, and the combined power of exclusion, CPE, was 0.999999989. For the Ecuadorian population, the combined random match probability was 1.02 × 10^−40^, and the combined power of exclusion was 0.999999978. The proposed method is intended to become an alternative to human identification based on differences in the length of STR loci.

## Simple Summary

For many decades, human identification by DNA has used the difference in the length of certain loci in the human genome—the so-called fragment analysis. Their main drawback is that they are too long, which interferes with the analysis if the DNA is damaged by environmental factors (rotting, sunlight, or chemical agents). Another drawback of using these regions for human identification is their high mutability, which leads to errors in determining kinship. The interest of forensic researchers in short polymorphisms like InDels has been attracted because of their potential advantages, such as low mutation rates and improved application in the analysis of degraded samples. We selected 99 InDels from the entire human genome in such a way as to create a universal set of markers that have the same identification potential regardless of the population in which it is used. In order to experimentally verify the correctness of our calculations, we examined two populations located on different continents: Russian and Ecuadorian. The results confirmed the correctness of our choice of markers. In this paper, we propose a set of markers and a biochip method for their genotyping which can be used for human identification in any population.

## 1. Introduction

For more than 30 years, the most frequently used DNA markers in human identification have been short tandem repeats (STRs). A significant disadvantage of STR markers is their large size (150–450 bp), which often leads to failure in the analysis of degraded DNA samples. Another drawback is due to the high mutation rate in STR loci (10^−2^–10^−3^ [1,2]), which creates the risk of errors in kinship determination.

To overcome the limitations of STRs, single nucleotide polymorphisms (SNPs) were proposed in 1993 [3]. They represent the most common and shortest type of genomic polymorphism and have a significantly lower mutation rate than STRs: 2.5 × 10^−8^ [4]. However, due to the biallelic nature of SNPs, to obtain a comparable discriminating potential, their number should be 2.5–4 times greater than STRs [5]. This requires complex methodological solutions that were unavailable before, including highly sensitive and highly multiplex PCR along with suitable genotyping methods. Nevertheless, the founder of DNA “fingerprinting”, Alec Jeffries, in his review publication dedicated to the 20th anniversary of genomic fingerprinting, pins his hopes on SNPs [6]. 

The type of polymorphisms of interest to forensic science is represented not only by SNPs but also by short InDels, which are practically equivalent in terms of size, prevalence in the genome, and mutability. Taking into account that the most promising method for their genotyping is hybridization analysis of amplified fragments, it is preferable to use short InDels, since discrimination of InDel alleles is more reliable than SNP due to more pronounced differences in the melting temperature of perfect and mismatch duplexes.

The only commercial forensic kit for InDel genotyping known to us is DIPplex^®^ (Qiagen, Hilden, Germany), intended for genotyping a panel of 30 polymorphisms by means of capillary electrophoresis [7]. About a hundred scientific publications devoted to the testing of DIPplex^®^ in various populations of the world testify to the demand for this area in forensic science, primarily due to the facility of genotyping degraded samples (the average amplicon length in DIPplex^®^ is 114 bp). However, it is not widely used, and no country in the world has used it to create a national genetic database. Apparently, this is due to the insufficiency of the identifying potential (CMP ≤ 2.83 × 10^−13^) and the low reliability of establishing kinship (combined probability of exclusion, CPE = 0.998).

Other works devoted to the creation of panels of biallelic markers, unfortunately, remained only scientific achievements. This is due to the presence of one or more significant drawbacks for implementation in practice: insufficient identifying potential or population specificity of alleles, or low sensitivity of the genotyping method [8,9,10,11,12,13,14,15].

The reason why none of the developments have been brought to implementation at the national level in any country, in our opinion, is complex. It lies in the absence of both a universal panel of biallelic polymorphisms with sufficient identifying potential in all world populations, and a method of its multiplex genotyping with the characteristics required in expert practice. In the present work, we attempted to solve this problem. When forming the panel of markers, we took into account the experience of previous work and the requirements of the modern forensic scientific community for the analytical capabilities of genetic identification systems. As a method for genotyping the formed panel of markers, we used an original approach developed at the IMB RAS, which is multiplex PCR followed by hybridization analysis on hydrogel biological microchips. 

The first aim of the work is to form a universal panel of markers and develop a tool for its genotyping that would meet current forensic criteria. The choice of InDels was made in such a way that their minor allele frequency in five superpopulations (EUR, EAS, SAS, AFR, and AMR) was at least 0.3. Based on the allele frequencies given in the 1000 Genomes Project, this provides a combined random match probability (CMP) of the order of 10^−41^. To confirm the versatility of the InDel panel, it is necessary to evaluate allele frequencies in populations that are phylogenetically distant from each other. For this purpose, here we explored Russian (N = 201) and Ecuadorian (N = 191) populations. We are aware that the Ecuadorian population is predominantly mixed, but it was chosen because it is the most phylogenetically distant from the Russian population of those available to us. The obtained results confirmed the expected analytical performance of the developed instrument, which we named ChipInDel99^®^.

## 2. Materials and Methods

Selection of highly informative InDel markers. InDels for this study were selected from the 1000 Genomes Project database, hosted on the FTP server of the Ensemble genome browser. At the first stage, the following criterion was set: the InDel should have a minor allele frequency ≥ 0.30 for all five populations (AFR, AMR, EUR, SAS, and EAS). As a result, a pool of 12,375 InDel polymorphisms was formed. In the second stage, the markers that were in the context of tandem repeats and polynucleotides were excluded, which reduced the pool to 1395. In the third stage, to reduce problems in the design of PCR primers, candidates containing tandem repeats and polynucleotides in the flanking region (±50 bp from InDel) or representing AT- or GC-rich regions were removed from the list of candidates. The remaining applicants were divided into clusters based on their location on the chromosome, and at the final stage were selected taking into account the uniqueness of flanking sequences by BLAST and the minimum distance to the next polymorphism of more than 3 million bp. The final list consisted of 99 polymorphisms and can be found in Supplementary Table S1. The nomenclature of polymorphisms and their coordinates are given according to the GRCh38.p13 assembly. 

DNA samples. A total of 392 unrelated individuals were analyzed in this study. Among them, 201 (100 male and 101 female) were from the Russian population (Moscow, Moscow region, St. Petersburg), and 191 (83 male and 108 female) were randomly selected from the city of Quito, Ecuador. All samples included in this study have been multiply coded and genotyped in anonymous form. Liquid saliva samples were collected using the Labtub kit (Lingen Precision Medical Products Co., Ltd., Shanghai, China) and stored at room temperature until extraction according to the manufacturer’s recommendations. Genomic DNA was extracted from saliva samples using LumiPure Genomic DNA Blood and Buccal Kit (Lumiprobe, Lumiprobe, Germany). The present study was conducted in accordance with the ethical standards of the 1975 Declaration of Helsinki, as revised in 2008, and all participants signed written informed consent before their recruitment in the study. The study was approved by the Bioethics Committee of the Engelhardt Institute of Molecular Biology, Russian Academy of Sciences (protocol 1 from 4 March 2024).

Multiplex. During the selection of PCR primers, the main goal was to minimize the size of the amplicon. The size of InDel loci in the multiplex system is listed in Supplementary Table S1, the mean value was 72 bp. To optimize the PCR conditions, several DNA samples were used at a concentration of 0.5 and 0.05 ng/µL each. After achieving reliable amplification by multiplex PCR, samples of the Russian and Ecuadorian populations were genotyped. A DNA sample (3 ng/µL) and deionized water were used as positive and negative controls, respectively. Several samples were diluted to 0.05 ng/µL to roughly estimate the sensitivity of the genotyping method.

PCR conditions. PCR was performed in 25 μL of the following composition: PCR buffer with HotTaqMulti polymerase, 4 units (Asfogen, Russia), 5 mM MgSO_4_, 0.2 mM of each of the dNTPs (Sibenzym, Novosibirsk, Russia), primer mixture, 200 pmol of Cy5-TCATTGGATCTCATTA universal primer, 0.05–50 ng of genomic DNA. Amplification was carried out in 0.2 mL PCR tubes on a SpeedCycler (AnalytikJena, Jena, Germany) with the following conditions: 95 °C for 2 min and 50 cycles in the first stage (95 °C for 20 s, 65 °C for 30 s, 66 °C for 30 s, 69 °C for 40 s), then 40 cycles in the second stage (95 °C—20 s, 56 °C—30 s, 72 °C—30 s). 

Biochip design. The biochip was manufactured according to the scheme shown in Supplementary Figure S1a. Allele-specific oligonucleotide probes were plotted from left to right in the order of their InDels in the genome (from the beginning of the first chromosome to the end of the 22 chromosome). Each polymorphism on the biochip corresponds to a pair of vertically arranged cells: the upper cell contains the probe for the allele with an insertion, and the lower cell contains a probe for the allele with a deletion. All oligonucleotides were produced by Lumiprobe (Germany). The fabrication of hydrogel biochips was carried out on Qarray2 (Genetix, New Milton, UK) in dust-free cleanrooms, according to the original technology of the IMB RAS as previously described [16], with minor changes.

Before applying hydrogel elements of biochips, the substrates were coated with a thin layer of poly(ethylene-co-propylene-co-5-methylene-2-norbornene) by the spin-coating method as described in [17]. The polymer coating improves the binding of the hydrogel elements of the biochip to the substrate.

Hybridization. The 30 µL chamber of the biochip was filled with a mixture of the following composition: 7.5 µL formamide, 7.5 µL 20× SSPE, and 15 µL PCR product. After incubation (10 h, 37 °C) and washing (10 min in 1× SSPE at room temperature), the biochips were washed with distilled water, dried with compressed air, placed in a portable analyzer “Picodetect” (OOO BIOCHIP-IMB, Moscow, Russia), and fluorescence was registered with an exposure of 0.5–2 s. Supplementary Figure S1b–e shows examples of such fluorescent images for four DNA samples. Image analysis was performed using ImaGel 2.0 and ImaGelStudio 4.0 software (IMB RAS). The result of the automatic processing of each biochip was a text file with the genotype of the sample.

Data processing. Allele frequencies and Hardy–Weinberg equilibrium were estimated using the online program https://gene-calc.pl/hardy-weinberg-page (accessed on 20 February 2023). The calculation of the key forensic characteristics of the InDel panel (MP, PD, CPD, PE, and CPE) was carried out in the program PowerStats v12.xls (Promega Corp., Madison, WI, USA) according to well-known formulas [18].

## 3. Results

### 3.1. InDel Selection

The basic parameter that determines the discriminating potential of any biallelic polymorphism is the minor allele frequency, MAF, and its ideal value for this task is 0.5. Due to the absence of the required number of polymorphisms with such characteristics simultaneously for all world human superpopulations, a compromise range of MAF must be found that will correspond to a certain pool from which further selection can be made according to second-order criteria. The less strictly the MAF value is set, the more choice of markers the researcher has, but the less the final discriminating potential will be for the panel. In other similar works, the authors set the MAF ≥ 0.20 [11,19] or less [20]. We tried to select markers that meet more stringent criteria: MAF ≥ 0.30.

When planning work on the InDels selection, the idea was that, while maintaining the specified MAF for all five superpopulations, the discriminating potential would not significantly decrease for more local subpopulations to which the developed method can potentially be applied. This will make the method universal on a global scale. When selecting candidate markers, we used data from the 1000 Genomes Project on allele and genotype frequencies in AFR (*n* = 661), AMR (*n* = 347), EUR (*n* = 503), SAS (*n* = 489), and EAS (*n* = 504). After the formation of the primary pool of 12,375 InDels, selected according to the MAF ≥ 0.30 criterion, we eliminated polymorphisms whose genotyping by hybridization analysis would be technically difficult or impossible. As a result of sequential selection according to the above criteria, a panel of 99 InDels was formed. 

The MAF of the markers (Supplementary Table S1) for the five considered populations ranged from 0.301 to 0.499. The average frequency of all minor alleles in each population was 0.392–0.400. The expected values of combined random match probability differ very little between the five superpopulations and are in the range of 1.44 × 10^−41^–3.68 × 10^−41^ (Supplementary Table S1), which confirms the validity of our approach. 

### 3.2. Characteristics of the InDel Set

The panel includes 99 InDels located on chromosomes 1–22 and a sex marker located in the amelogenin gene. The distance between adjacent markers varies from 3.3 Mbp to 113.6 Mbp (the average distance is 24 Mbp), which excludes possible linked inheritance of markers. The size of InDel polymorphisms varies from 1 to 18 nucleotides (mean 4.3, median 3). The length of the amplified loci ranged from 44 to 108 bp, the mean and median values coincided and amounted to 72 bp for loci containing the Del allele and 76 bp for loci containing the In allele. 

### 3.3. Genotyping Method 

The genotyping protocol contains four stages: DNA extraction, amplification, hybridization, and registration of results. DNA extraction can be performed in any manner suitable for the biomaterial. The method has been tested in a wide range of concentrations and does not require adjustment to acceptable values, unlike STR-typing. Amplification was performed in a single multiplex PCR. For all the studied samples, fluorescence hybridization patterns of microarrays were obtained and analyzed. Four examples of such patterns are shown in Supplementary Figure S1b–d). 

### 3.4. Population Data and Forensic Utility of the 99 InDels

To verify the characteristics of the formed panel, we considered it necessary to evaluate the allelic frequencies of its markers in different populations. In this regard, we chose the two most phylogenetically remote populations at our disposal: Russian and Ecuadorian. 

The data obtained are summarized in Supplementary Table S2. As can be seen from the results, the vast majority of markers met the specified criteria. In the Russian population studied in this work, the average frequency of minor alleles was 0.385, and the average observed heterozygosity, Ho = 0.470 (0.338–0.687). In two polymorphisms, the MAF was slightly less than the preestablished value of 0.3: CID.04-057, 0.254 and CID.04–112, 0.266 (Supplementary Table S2). In the Ecuadorian population, the average frequency of minor alleles was 0.389, and the average observed heterozygosity, Ho = 0.466 (0.358–0.579). In seven polymorphisms, the MAF was slightly less than the preestablished value: CID.02–216, 0.287, CID.05–095, 0.291 CID.06–125, 0.298, CID.10–071, 0.298 CID.11–083, 0.279, CID.12–090, 0.237, and CID.20–056, 0.283 (Supplementary Table S2). 

Based on the obtained data, we determined the MP value for each InDel (Supplementary Table S2) and also evaluated the main forensic characteristics of the entire panel for both populations. For the Russian population, the combined random match probability, CMP, was 2.09 × 10^−40^, and the combined power of exclusion, CPE, = 0.999999989. and for Ecuadorian 1.02 × 10^−40^ and 0.999999978, respectively. 

## 4. Discussion

There are two main indicators for all systems of human genetic identification that determine their analytical power: the CMP specifying the discrimination power of the system, and the CPE that evaluates the applicability of the tool in kinship analysis. The CMP value of the ChipInDel99 panel for both studied populations was about 10^−40^, and the CPE was ≈ 0.9999999. 

Comparing the CMP obtained for ChipInDel99 set of markers with that of the most famous STR-systems: 13-locus CODIS—2.34 × 10^−15^; Identifiler^®^ (Applied Biosystems, Waltham, MA, USA)—5.93 × 10^−18^; PowerPlex16^®^ (Promega, Madison, WI, USA)—2.43 × 10^−18^; AmpFlSTR^®^ NGM™ (Applied Biosystems, Waltham, MA, USA)—1.12 × 10^−19^; and New FBI core—6.28 × 10^−30^ [21], it can be seen that the proposed panel has 10 orders of magnitude higher discriminating potential than even New FBI core—the most informative panel used in practice. This redundancy in the ChipInDel99 discriminating potential provides an advantage in the examination of complex samples for which incomplete profiles are obtained.

CPE for the most applied currently available commercial multiplex kits: Identifiler^®^—0.999996 [22]; PowerPlex16^®^—0.9994 [23]; and AmpFlSTR^®^ NGM™—0.9999998 [24] is also inferior to that of the ChipInDel99. Thus, the ChipInDel99 kit was observed to be highly informative in two phylogenetically distant populations which indicates that the selected polymorphisms correspond to the criteria specified during selection and suggests that the proposed panel will be universal for most world populations. 

Special efforts have been made to create conditions for the introduction of the method into practice and to reduce the risk of contamination. Namely, previously, kits based on biochip technology had a number of disadvantages that limited their widespread use. In particular, due to the need to obtain a single-stranded amplicon for hybridization, the protocol used a two-stage nested PCR with the transfer of amplicons from the symmetric PCR stage to the asymmetric one, which required a separate laboratory area and created a serious risk of contamination. Thanks to the use of a previously published modification (LATE)-PCR [25], it was possible to combine the symmetric and asymmetric PCR stages at the amplification process. Thus, the study protocol was simplified as much as possible, reduced to the “one sample—one tube—one biochip” scheme and freed from the intermediate stages of amplicon transfer. 

In addition to discrimination power, the most important quality of practical forensic methods is the ability to genotype degraded samples. The key to success in dealing with fragmented DNA is the minimization of amplicons when designing primers. For PCR followed by hybridization, the minimum fragment length that can be obtained depends on the nucleotide composition of the locus, and, ideally, is 40–50 bp. We succeeded in reaching an average locus length of 72 bp, which provides the method with a fundamental advantage in the study of degraded DNA. However, experimental verification on degraded samples was not included in the plan of this work.

## 5. Conclusions

Currently, human genetic identification is entirely based on STR: equipment, databases, and results interpretation methods are focused on this type of marker. However, as mentioned in the introduction, strategic STRs are inferior to SNPs and InDels in length and variability, and this disadvantage is fundamental; due to the nature of STR polymorphisms, it cannot be overcome by technical means. We do not reject the use of STR in forensic science; we propose using an alternative method that allows solving those problems that STR cannot solve. The request for an identification method using short biallelic markers arose long ago but was initially not implemented due to the lack of a suitable genotyping tool. In this paper, we have achieved preliminary success in creating a practical tool for solving forensic genetic examination problems at a new level: we have proposed a panel of markers that are versatile enough to be used in forensic genetic examination and become the basis for the formation of a national genetic information database. The choice of the method in favor of the technology of low-density biological microchips is the most appropriate for the tasks set since it ensures the placement of the required number of oligonucleotide probes and does not require complex sample preparation protocols, expensive equipment, and consumables, which creates the prerequisites for its successful implementation. To assess the applicability of the genotyping method for this panel, we plan to perform validation studies according to the SWGDAM guidelines.

## Data Availability

The data presented in this study are available on request from the corresponding author due to the restrictions specified in the informed consent: the DNA donors who participated in the study permit the publication of the data only in an anonymized form that does not allow their identity to be identified.

## Supplementary Materials

The following supporting information can be downloaded at: https://www.mdpi.com/article/10.3390/biology13120993/s1, Table S1: A description, location, and distribution of 99 markers included in the ChipInDel99 panel. Minor allele frequencies in five superpopulations are given from the 1000 Genome Project data. Table S2: Allele and genotype frequencies, random match probability for 99 InDels in Russian and Ecuadorian populations, combined random match probability, and combined power of exclusion of the entire set of markers for both populations. Figure S1: Scheme of the arrangement of DNA probes on the biochip ChipInDel99 (a) and examples of fluorescent images of the biochip obtained as a result of genotyping of four unrelated male (b,c) and female (d,e) DNA samples. In the scheme, the numbers above each pair of cells correspond to the number of the polymorphism from Supplementary Table S1. Designations inside cells correspond to alleles of InDels.
