# Supplementary material for: The Universal Set of 99 InDel Markers for Human Identification
Source: Biology (Basel). 2024 Nov 29;13(12):993. doi: 10.3390/biology13120993 (PMC11726970; doi:10.3390/biology13120993)
Supplement: Supplementary file 1 [file biology-13-00993-s001.zip › Supplementary Table S1.pdf]

Table S1. A description, location and distribution of 99 markers included in the *ChipInDel99* panel. Minor allele frequencies in five super populations are given from the *1000 Genome Project* data.

#	RS number	*ChipInDel* loci code	Location	Alleles	MA*	L**, bp	Minor allele frequency (MAF) in super populations, F_o				
							EAS	EUR	AMR	SAS	AFR
**1**	rs10617833	CID.01-042	1:41899280	TCC/-	Del	55	0.398	0.374	0.401	0.331	0.476
**2**	rs11394524	CID.01-120	1:119994503	G/-	Del	66	0.414	0.412	0.496	0.318	0.316
**3**	rs72082809	CID.01-155	1:154776010	GTAA/-	Del	65	0.336	0.498	0.326	0.404	0.495
**4**	rs35379245	CID.01-171	1:170771419	AG/-	In	62	0.495	0.327	0.376	0.482	0.398
**5**	rs71107464	CID.01-192	1:191712777	AAT/-	Del	68	0.340	0.408	0.421	0.473	0.489
**6**	rs66765111	CID.01-208	1:207842739	ATTTCAGTTTT CTTCGAG/-	Del	65	0.332	0.381	0.318	0.320	0.473
**7**	rs35126101	CID.01-223	1:223222802	TG/-	Del	64	0.369	0.319	0.349	0.410	0.473
**8**	rs34450453	CID.01-240	1:239878518	TGG/-	In	84	0.400	0.426	0.454	0.445	0.325
**9**	rs10693974	CID.02-012	2:11733545	ACTC/-	In	61	0.436	0.388	0.484	0.387	0.471
**10**	rs66610049	CID.02-036	2:36026644	CTGT/-	Del	74	0.448	0.373	0.388	0.343	0.320
**11**	rs57616890	CID.02-043	2:42762877	ATA/-	Del	69	0.346	0.374	0.324	0.454	0.405
**12**	rs72339906	CID.02-080	2:80191212	AG/-	Del	58	0.431	0.435	0.454	0.373	0.394
**13**	rs5832955	CID.02-102	2:101846803	G/-	In	71	0.405	0.427	0.440	0.483	0.309
**14**	rs111665673	CID.02-216	2:215632035	AGAG/-	In	76	0.343	0.398	0.308	0.427	0.375
**15**	rs57993635	CID.02-232	2:232275593	C/-	In	76	0.419	0.423	0.347	0.390	0.320
**16**	rs35926392	CID.03-016	3:16145615	GC/-	Del	66	0.362	0.474	0.441	0.385	0.372
**17**	rs139738417	CID.03-031	3:30759018	CATAAGC/-	In	75	0.321	0.354	0.349	0.402	0.451
**18**	rs57495895	CID.03-082	3:82011691	G/-	Del	70	0.456	0.393	0.406	0.310	0.343
**19**	rs35291341	CID.03-100	3:99843746	CT/-	In	64	0.350	0.367	0.432	0.465	0.492
**20**	rs35464243	CID.03-107	3:106930411	CAC/-	In	72	0.477	0.330	0.363	0.445	0.359
**21**	rs10540628	CID.03-124	3:123992597	AC/-	Del	67	0.330	0.424	0.455	0.308	0.465
**22**	rs59170274	CID.04-027	4:26821887	C/-	Del	65	0.371	0.405	0.483	0.468	0.350
**23**	rs34184705	CID.04-045	4:44871992	G/-	Del	59	0.310	0.469	0.334	0.492	0.433
**24**	rs56281469	CID.04-057	4:57360344	CT/-	In	66	0.476	0.312	0.484	0.368	0.387
**25**	rs35932180	CID.04-086	4:85828903	CT/-	In	47	0.366	0.351	0.473	0.353	0.470
**26**	rs72273695	CID.04-113	4:112718708	ATCTGCATTG/-	Del	106	0.421	0.302	0.326	0.456	0.306
**27**	rs70938781	CID.04-138	4:138358907	CCTACCTTGTA GCT/-	In	62	0.418	0.490	0.448	0.403	0.409
**28**	rs3067059	CID.04-178	4:178345624	ACTTG/-	In	77	0.368	0.331	0.316	0.373	0.328
**29**	rs61235135	CID.05-003	5:3358309	C/-	Del	62	0.396	0.379	0.395	0.348	0.382
**30**	rs34159280	CID.05-014	5:13705943	CTTCACAAGA/-	Del	72	0.393	0.393	0.405	0.452	0.330
**31**	rs142490175	CID.05-064	5:63882726	GAAAG/-	Del	96	0.336	0.340	0.380	0.404	0.413
**32**	rs138124389	CID.05-087	5:86744866	TAGACACTGC AGACTAC/-	In	70	0.465	0.416	0.412	0.449	0.433
**33**	rs34990478	CID.05-095	5:95002440	CAT/-	Del	65	0.319	0.382	0.370	0.365	0.435
**34**	rs6149192	CID.05-115	5:114944029	GACCCTAAAC AGAG/-	Del	72	0.478	0.489	0.402	0.454	0.415
**35**	rs71621395	CID.05-119	5:118963126	AAC/-	Del	85	0.487	0.441	0.379	0.491	0.327
**36**	rs66881681	CID.05-132	5:131813412	AGG/-	Del	77	0.384	0.333	0.353	0.431	0.470
**37**	rs10701589	CID.05.140	5:140036116	TGAT /-	In	71	0.452	0.431	0.363	0.451	0.384
**38**	rs3832355	CID.05-150	5:150038428	ACTC/-	Del	65	0.331	0.417	0.494	0.406	0.473
**39**	rs10560231	CID.05-180	5:179630655	ATG/-	Del	92	0.471	0.412	0.460	0.411	0.396
**40**	rs35948562	CID.06-007	6:7387821	CA/-	In	73	0.450	0.385	0.463	0.366	0.497
**41**	rs56071028	CID.06-016	6:15988568	C/-	In	73	0.455	0.404	0.415	0.363	0.426
**42**	rs10529292	CID.06-031	6:31409184	ACA/-	In	64	0.304	0.330	0.427	0.312	0.470
**43**	rs68133212	CID.06-072	6:71745181	TCT/-	Del	76	0.384	0.347	0.402	0.408	0.346
**44**	rs11267328	CID.06-078	6:77750524	ACTCAGTTCAT GA/-	Del	69	0.325	0.447	0.346	0.344	0.465
**45**	rs3831872	CID.06-088	6:88183335	CAGTTAGAAG GAACACT/-	In	61	0.306	0.346	0.487	0.390	0.435
**46**	rs56343259	CID.06-096	6:96279205	GCCC/-	In	56	0.347	0.354	0.366	0.397	0.427
**47**	rs141141409	CID.06-125	6:124946472	GTT/-	In	64	0.411	0.358	0.318	0.303	0.414
**48**	rs57057239	CID.06-135	6:135024728	GAAT/-	Del	95	0.442	0.359	0.356	0.447	0.356
**49**	rs6149835	CID.06-144	6:143819409	TTGAACAAAG TCC/-	Del	76	0.482	0.339	0.440	0.359	0.478
**50**	rs11278298	CID.06-159	6:158930905	TCATTCAG/-	Del	71	0.364	0.366	0.405	0.491	0.487
**51**	rs35345873	CID.07-010	7:10352781	G/-	In	85	0.379	0.407	0.334	0.408	0.356
**52**	rs68111476	CID.07-023	7:23027696	TTTGCCTGATA ACC/-	Del	66	0.468	0.363	0.329	0.347	0.358
**53**	rs1610906	CID.07-082	7:81725951	TAT/-	In	72	0.393	0.437	0.450	0.380	0.346
**54**	rs57943214	CID.07-097	7:96765416	GTATTTG/-	In	82	0.468	0.351	0.412	0.401	0.470

**55**	rs3216282	CID.07-126	7:126081845	AGAT/-	Del	74	0.379	0.318	0.392	0.369	0.340
**56**	rs35575707	CID.07-140	7:140101500	AG/-	Del	68	0.495	0.399	0.370	0.406	0.486
**57**	rs35146459	CID.08-002	8:1788862	G/-	Del	55	0.377	0.385	0.383	0.332	0.338
**58**	rs35604660	CID.08-059	8:59055597	GT/-	In	81	0.342	0.411	0.458	0.355	0.378
**59**	rs34067160	CID.08-065	8:65072454	AA/-	In	96	0.471	0.427	0.435	0.418	0.392
**60**	rs35679778	CID.08-104	8:103906808	C/-	In	71	0.432	0.398	0.486	0.473	0.456
**61**	rs11323264	CID.08-127	8:126654202	G/-	Del	44	0.475	0.414	0.365	0.443	0.341
**62**	rs6150855	CID.08-141	8:140579696	AGGCTGAGCAG/-	Del	62	0.357	0.356	0.326	0.391	0.414
**63**	rs56962291	CID.09-072	9:72269223	CTCCT/-	In	81	0.486	0.385	0.432	0.460	0.315
**64**	rs34499887	CID.09-087	9:86660018	G/-	Del	50	0.493	0.463	0.352	0.465	0.365
**65**	rs59923204	CID.09-109	9:108837145	C/-	Del	54	0.409	0.347	0.373	0.457	0.362
**66**	rs55952736	CID.09-117	9:117187806	TAGC/-	In	76	0.329	0.335	0.378	0.301	0.379
**67**	rs71953876	CID.09-135	9:135380181	CCAC/-	Del	63	0.352	0.305	0.330	0.319	0.469
**68**	rs10552811	CID.10-071	10:70563084	GC/-	Del	79	0.302	0.467	0.320	0.336	0.405
**69**	rs5786663	CID.10-085	10:84613642	TA/-	Del	70	0.391	0.394	0.377	0.398	0.320
**70**	rs140576359	CID.10-118	10:117733203	CCGATATT/-	Del	71	0.409	0.412	0.414	0.373	0.429
**71**	rs10544160	CID.10-128	10:128080091	TA/-	Del	74	0.349	0.410	0.428	0.351	0.303
**72**	rs10583916	CID.11-006	11:5870839	TG/-	Del	94	0.460	0.369	0.314	0.372	0.362
**73**	rs113764748	CID.11-020	11:19528202	TAGC/-	Del	71	0.435	0.452	0.415	0.411	0.327
**74**	rs74933486	CID.11-083	11:83237025	CA/-	Del	86	0.458	0.458	0.313	0.461	0.437
**75**	rs35847449	CID.11-094	11:94191207	C/-	In	57	0.403	0.373	0.406	0.412	0.335
**76**	rs56221618	CID.11-111	11:111065696	AAG/-	Del	61	0.403	0.356	0.346	0.471	0.499
**77**	rs11394480	CID.11-135	11:134778510	G/-	In	47	0.465	0.349	0.463	0.301	0.408
**78**	rs10533848	CID.12-013	12:12861586	TC/-	Del	77	0.450	0.445	0.437	0.465	0.312
**79**	rs10689649	CID.12-090	12:89969919	ACTC/-	In	93	0.362	0.380	0.303	0.433	0.424
**80**	rs56392226	CID.13-040	13:40028153	AATGC/-	Del	81	0.365	0.412	0.424	0.442	0.359
**81**	rs59502417	CID.13-087	13:87305513	TGTC/-	Del	90	0.313	0.406	0.431	0.346	0.313
**82**	rs55812371	CID.14-056	14:56159616	ACATGTTCCA/-	In	69	0.367	0.343	0.310	0.427	0.307
**83**	rs3085775	CID.14-091	14:91332415	AGTG/-	In	87	0.407	0.437	0.474	0.368	0.329
**84**	rs67320356	CID.15-067	15:66925590	GAGTTT/-	Del	70	0.349	0.359	0.398	0.477	0.368
**85**	rs3995812	CID.15-098	15:97502107	TTAC/-	In	61	0.401	0.334	0.401	0.307	0.307
**86**	rs34436424	CID.16-012	16:11820079	GC/-	Del	80	0.319	0.499	0.304	0.356	0.467
**87**	rs3841767	CID.16-024	16:24136639	CC/-	Del	68	0.433	0.415	0.455	0.340	0.428
**88**	rs34816330	CID.16-076	16:76041094	C/-	Del	60	0.330	0.485	0.424	0.433	0.421
**89**	rs5821134	CID.17-057	17:56529684	ACTC/-	In	78	0.308	0.454	0.318	0.446	0.478
**90**	rs141730340	CID.17-081	17:80625313	CCTGTGCAGCG/-	Del	95	0.419	0.311	0.304	0.348	0.363
**91**	rs35452393	CID.18-028	18:27684881	AT/-	In	56	0.472	0.490	0.318	0.458	0.432
**92**	rs33990768	CID.18-048	18:48155140	G/-	Del	58	0.362	0.481	0.375	0.410	0.441
**93**	rs112877961	CID.19-029	19:29495432	C/-	Del	59	0.445	0.393	0.411	0.468	0.492
**94**	rs35894782	CID.20-020	20:20384749	AATAG/-	Del	78	0.366	0.410	0.369	0.348	0.440
**95**	rs73622051	CID.20-038	20:37610825	TTTA/-	Del	65	0.430	0.347	0.370	0.340	0.347
**96**	rs34198404	CID.20-056	20:56104372	GTG/-	Del	76	0.446	0.451	0.336	0.362	0.478
**97**	rs10583984	CID.21-039	21:39042013	AG/-	Del	85	0.415	0.384	0.441	0.318	0.315
**98**	rs35282131	CID.21-044	21:43822529	ACAT/-	Del	67	0.457	0.370	0.344	0.389	0.466
**99**	rs60228580	CID.22-033	22:33384984	G/-	In	76	0.358	0.401	0.411	0.424	0.425
	The average MAF value						**0.399**	**0.394**	**0.392**	**0.398**	**0.400**
	Estimated Combined Match Probability (CMP) for 99 markers, $\times10^{-41}$						**2.652**	**3.685**	**3.304**	**1.435**	**3.145**

* MA – minor allele, is such for all super populations

** L – length of amplicon in *ChipInDel99* multiplex set; specified for PCR product containing the Del allele

F_o – observed frequency
