# Supplementary material for: The Universal Set of 99 InDel Markers for Human Identification
Source: Biology (Basel). 2024 Nov 29;13(12):993. doi: 10.3390/biology13120993 (PMC11726970; doi:10.3390/biology13120993)
Supplement: Supplementary file 1 [file biology-13-00993-s001.zip › Supplementary Table S2.pdf]

Table S2. Allele and genotype frequencies, match probability for 99 indels in Russian and Ecuadorian populations, combined match probability and combined power of exclusion of the entire set of markers for both populations.

	Russian population (N=201)						Ecuadorian population (N=191)					
	In/In	In/Del	Del/Del	In	Del	MP	In/In	In/Del	Del/Del	In	Del	MP
**CID.01-042**	0.443	0.388	0.169	0.637	0.363	0.375	0.376	0.500	0.124	0.626	0.374	0.407
**CID.01-120**	0.179	0.408	0.413	0.383	0.617	0.369	0.247	0.579	0.174	0.537	0.463	0.427
**CID.01-155**	0.210	0.580	0.210	0.500	0.500	0.425	0.450	0.419	0.131	0.660	0.340	0.395
**CID.01-171**	0.144	0.448	0.408	0.368	0.632	0.388	0.102	0.471	0.428	0.337	0.663	0.415
**CID.01-192**	0.353	0.483	0.164	0.595	0.405	0.385	0.277	0.487	0.236	0.521	0.479	0.370
**CID.01-208**	0.455	0.440	0.105	0.675	0.325	0.412	0.458	0.442	0.100	0.679	0.321	0.415
**CID.01-223**	0.458	0.433	0.109	0.674	0.326	0.409	0.437	0.453	0.111	0.663	0.337	0.408
**CID.01-240**	0.191	0.437	0.372	0.410	0.590	0.366	0.217	0.509	0.274	0.471	0.529	0.381
**CID.02-012**	0.095	0.433	0.472	0.311	0.689	0.420	0.215	0.513	0.272	0.471	0.529	0.383
**CID.02-036**	0.338	0.473	0.189	0.575	0.425	0.374	0.387	0.440	0.173	0.607	0.393	0.373
**CID.02-043**	0.483	0.388	0.129	0.677	0.323	0.400	0.446	0.452	0.102	0.672	0.328	0.414
**CID.02-080**	0.328	0.518	0.154	0.587	0.413	0.399	0.236	0.476	0.288	0.474	0.526	0.365
**CID.02-102**	0.217	0.455	0.328	0.444	0.556	0.362	0.209	0.503	0.288	0.461	0.539	0.379
**CID.02-216**	0.210	0.490	0.300	0.455	0.545	0.374	0.095	0.384	0.521	0.287	0.713	0.428
**CID.02-232**	0.189	0.393	0.418	0.386	0.614	0.365	0.120	0.424	0.455	0.332	0.668	0.402
**CID.03-016**	0.338	0.493	0.169	0.585	0.415	0.386	0.487	0.402	0.111	0.688	0.312	0.411
**CID.03-031**	0.194	0.507	0.299	0.448	0.552	0.384	0.153	0.524	0.323	0.415	0.585	0.402
**CID.03-082**	0.368	0.458	0.174	0.597	0.403	0.375	0.324	0.389	0.286	0.519	0.481	0.339
**CID.03-100**	0.154	0.438	0.408	0.373	0.627	0.382	0.263	0.437	0.300	0.482	0.518	0.350
**CID.03-107**	0.109	0.388	0.503	0.303	0.697	0.415	0.115	0.508	0.377	0.369	0.631	0.413
**CID.03-124**	0.313	0.488	0.199	0.557	0.443	0.376	0.219	0.551	0.230	0.495	0.505	0.404
**CID.04-027**	0.338	0.508	0.154	0.592	0.408	0.396	0.277	0.524	0.199	0.539	0.461	0.391
**CID.04-045**	0.398	0.463	0.139	0.629	0.371	0.392	0.426	0.458	0.116	0.655	0.345	0.405
**CID.04-057**	0.085	0.338	0.577	0.254	0.746	0.455	0.316	0.453	0.232	0.542	0.458	0.358
**CID.04-086**	0.129	0.428	0.443	0.343	0.657	0.396	0.277	0.461	0.262	0.508	0.492	0.358
**CID.04-113**	0.537	0.393	0.070	0.734	0.266	0.448	0.288	0.534	0.178	0.555	0.445	0.400
**CID.04-138**	0.205	0.500	0.295	0.455	0.545	0.379	0.227	0.453	0.320	0.453	0.547	0.359
**CID.04-178**	0.090	0.512	0.398	0.346	0.654	0.429	0.095	0.476	0.429	0.333	0.667	0.420
**CID.05-003**	0.423	0.448	0.129	0.647	0.353	0.396	0.326	0.516	0.158	0.584	0.416	0.397
**CID.05-014**	0.448	0.477	0.075	0.687	0.313	0.434	0.361	0.513	0.126	0.618	0.382	0.410
**CID.05-064**	0.403	0.458	0.139	0.632	0.368	0.391	0.406	0.476	0.118	0.644	0.356	0.406
**CID.05-087**	0.179	0.507	0.314	0.433	0.567	0.388	0.186	0.436	0.378	0.404	0.596	0.368
**CID.05-095**	0.378	0.453	0.169	0.604	0.396	0.377	0.471	0.476	0.053	0.709	0.291	0.451
**CID.05-115**	0.234	0.473	0.293	0.470	0.530	0.364	0.374	0.471	0.155	0.610	0.390	0.386
**CID.05-119**	0.408	0.453	0.139	0.634	0.366	0.391	0.293	0.518	0.188	0.552	0.448	0.390
**CID.05-132**	0.423	0.483	0.094	0.664	0.336	0.421	0.424	0.419	0.157	0.634	0.366	0.380
**CID.05.140**	0.184	0.522	0.294	0.445	0.555	0.393	0.141	0.508	0.351	0.395	0.605	0.401
**CID.05-150**	0.353	0.503	0.144	0.604	0.396	0.398	0.283	0.492	0.225	0.529	0.471	0.373
**CID.05-180**	0.308	0.463	0.229	0.540	0.460	0.362	0.380	0.484	0.136	0.622	0.378	0.397
**CID.06-007**	0.094	0.488	0.418	0.338	0.662	0.421	0.279	0.505	0.216	0.532	0.468	0.380
**CID.06-016**	0.159	0.483	0.358	0.400	0.600	0.387	0.147	0.521	0.332	0.408	0.592	0.403
**CID.06-031**	0.114	0.403	0.483	0.316	0.684	0.408	0.221	0.479	0.300	0.461	0.539	0.368
**CID.06-072**	0.463	0.418	0.119	0.672	0.328	0.403	0.386	0.466	0.148	0.619	0.381	0.388
**CID.06-078**	0.388	0.488	0.124	0.632	0.368	0.404	0.426	0.437	0.137	0.645	0.355	0.391
**CID.06-088**	0.159	0.403	0.438	0.361	0.639	0.379	0.262	0.482	0.257	0.503	0.497	0.366
**CID.06-096**	0.119	0.468	0.413	0.353	0.647	0.403	0.152	0.419	0.429	0.361	0.639	0.383
**CID.06-125**	0.090	0.485	0.425	0.333	0.668	0.424	0.079	0.440	0.482	0.298	0.702	0.432
**CID.06-135**	0.358	0.522	0.120	0.619	0.381	0.415	0.450	0.407	0.143	0.653	0.347	0.389
**CID.06-144**	0.468	0.413	0.119	0.674	0.326	0.403	0.342	0.489	0.168	0.587	0.413	0.385
**CID.06-159**	0.448	0.433	0.119	0.664	0.336	0.402	0.382	0.461	0.157	0.613	0.387	0.383
**CID.07-010**	0.149	0.483	0.368	0.391	0.609	0.391	0.121	0.384	0.495	0.313	0.687	0.407
**CID.07-023**	0.420	0.445	0.135	0.643	0.358	0.393	0.447	0.495	0.058	0.695	0.305	0.448
**CID.07-082**	0.219	0.517	0.264	0.478	0.522	0.385	0.246	0.461	0.293	0.476	0.524	0.359
**CID.07-097**	0.100	0.428	0.472	0.313	0.687	0.416	0.220	0.445	0.335	0.442	0.558	0.359
**CID.07-126**	0.482	0.437	0.081	0.701	0.299	0.430	0.340	0.440	0.220	0.560	0.440	0.358
**CID.07-140**	0.418	0.393	0.189	0.614	0.386	0.365	0.455	0.404	0.140	0.657	0.343	0.390
**CID.08-002**	0.324	0.537	0.139	0.592	0.408	0.413	0.330	0.487	0.183	0.573	0.427	0.379
**CID.08-059**	0.194	0.502	0.304	0.445	0.555	0.382	0.196	0.471	0.333	0.431	0.569	0.371
**CID.08-065**	0.189	0.438	0.373	0.408	0.592	0.367	0.265	0.497	0.238	0.513	0.487	0.374
**CID.08-104**	0.144	0.453	0.403	0.371	0.629	0.388	0.335	0.463	0.202	0.566	0.434	0.367
**CID.08-127**	0.373	0.448	0.179	0.597	0.403	0.372	0.380	0.452	0.169	0.605	0.395	0.377
**CID.08-141**	0.428	0.443	0.129	0.649	0.351	0.396	0.455	0.424	0.120	0.668	0.332	0.402
**CID.09-072**	0.104	0.473	0.423	0.341	0.659	0.413	0.137	0.456	0.407	0.365	0.635	0.392
**CID.09-087**	0.234	0.557	0.209	0.512	0.488	0.409	0.396	0.481	0.123	0.636	0.364	0.403

**CID.09-109**	0.473	0.368	0.159	0.657	0.343	0.384	0.497	0.366	0.136	0.681	0.319	0.400
**CID.09-117**	0.094	0.687	0.219	0.438	0.562	0.528	0.116	0.411	0.474	0.321	0.679	0.406
**CID.09-135**	0.418	0.458	0.124	0.647	0.353	0.400	0.447	0.452	0.101	0.673	0.327	0.414
**CID.10-071**	0.323	0.473	0.204	0.560	0.440	0.370	0.497	0.408	0.094	0.702	0.298	0.423
**CID.10-085**	0.443	0.403	0.154	0.644	0.356	0.382	0.422	0.503	0.075	0.674	0.326	0.437
**CID.10-118**	0.314	0.542	0.144	0.585	0.415	0.413	0.353	0.500	0.147	0.603	0.397	0.396
**CID.10-128**	0.343	0.483	0.174	0.585	0.415	0.381	0.293	0.513	0.194	0.550	0.450	0.387
**CID.11-006**	0.404	0.481	0.115	0.644	0.356	0.408	0.425	0.527	0.048	0.689	0.311	0.461
**CID.11-020**	0.264	0.542	0.194	0.535	0.465	0.401	0.332	0.479	0.189	0.571	0.429	0.375
**CID.11-083**	0.333	0.518	0.149	0.592	0.408	0.401	0.516	0.410	0.074	0.721	0.279	0.440
**CID.11-094**	0.100	0.502	0.398	0.351	0.649	0.421	0.168	0.478	0.353	0.408	0.592	0.382
**CID.11-111**	0.443	0.482	0.075	0.684	0.316	0.435	0.330	0.534	0.136	0.597	0.403	0.413
**CID.11-135**	0.179	0.403	0.418	0.381	0.619	0.369	0.131	0.492	0.377	0.377	0.623	0.401
**CID.12-013**	0.224	0.512	0.264	0.480	0.520	0.382	0.345	0.503	0.152	0.596	0.404	0.395
**CID.12-090**	0.104	0.473	0.423	0.341	0.659	0.413	0.058	0.358	0.584	0.237	0.763	0.473
**CID.13-040**	0.383	0.498	0.119	0.632	0.368	0.409	0.307	0.519	0.175	0.566	0.434	0.394
**CID.13-087**	0.443	0.448	0.109	0.667	0.333	0.409	0.242	0.558	0.200	0.521	0.479	0.410
**CID.14-056**	0.094	0.458	0.448	0.323	0.677	0.419	0.112	0.436	0.452	0.330	0.670	0.407
**CID.14-091**	0.174	0.498	0.328	0.423	0.577	0.386	0.272	0.492	0.236	0.518	0.482	0.372
**CID.15-067**	0.423	0.448	0.129	0.647	0.353	0.396	0.452	0.426	0.122	0.665	0.335	0.400
**CID.15-098**	0.099	0.488	0.413	0.343	0.657	0.418	0.173	0.471	0.356	0.408	0.592	0.379
**CID.16-012**	0.328	0.478	0.194	0.567	0.433	0.374	0.492	0.414	0.094	0.699	0.301	0.422
**CID.16-024**	0.338	0.537	0.125	0.607	0.393	0.419	0.298	0.534	0.168	0.565	0.435	0.402
**CID.16-076**	0.289	0.562	0.149	0.570	0.430	0.422	0.291	0.418	0.291	0.500	0.500	0.344
**CID.17-057**	0.219	0.492	0.289	0.465	0.535	0.374	0.102	0.406	0.492	0.305	0.695	0.418
**CID.17-081**	0.408	0.497	0.095	0.657	0.343	0.423	0.420	0.489	0.091	0.665	0.335	0.424
**CID.18-028**	0.343	0.463	0.194	0.575	0.425	0.370	0.168	0.424	0.408	0.380	0.620	0.375
**CID.18-048**	0.244	0.517	0.239	0.502	0.498	0.384	0.429	0.435	0.136	0.647	0.353	0.392
**CID.19-029**	0.393	0.507	0.100	0.647	0.353	0.422	0.389	0.511	0.100	0.645	0.355	0.422
**CID.20-020**	0.428	0.478	0.094	0.667	0.333	0.420	0.444	0.423	0.132	0.656	0.344	0.394
**CID.20-038**	0.438	0.453	0.109	0.664	0.336	0.409	0.444	0.438	0.119	0.663	0.338	0.402
**CID.20-056**	0.338	0.478	0.184	0.577	0.423	0.376	0.508	0.419	0.073	0.717	0.283	0.439
**CID.21-039**	0.368	0.507	0.125	0.622	0.378	0.409	0.304	0.513	0.183	0.560	0.440	0.389
**CID.21-044**	0.328	0.498	0.174	0.577	0.423	0.386	0.392	0.476	0.132	0.630	0.370	0.398
**CID.22-033**	0.169	0.488	0.343	0.413	0.587	0.384	0.199	0.445	0.356	0.421	0.579	0.364
**CMP**						**$2.09 \cdot 10^{-40}$**						**$1.02 \cdot 10^{-40}$**
**CPE**						**0.999999989**						**0.999999978**
